# Acid-sensing ion channel 1a contributes to the effect of extracellular acidosis on NLRP1 inflammasome activation in cortical neurons

**DOI:** 10.1186/s12974-015-0465-7

**Published:** 2015-12-30

**Authors:** Yu-Chan Wang, Wei-Zu Li, Yu Wu, Yan-Yan Yin, Liu-Yi Dong, Zhi-Wu Chen, Wen-Ning Wu

**Affiliations:** Department of Pharmacology, Key Laboratory of Anti-inflammatory and Immunopharmacology, School of Basic Medical Sciences, Anhui Medical University, Hefei, 230032 China

**Keywords:** Inflammasome, Acidosis, ASICs, BK channels, Cortical neurons

## Abstract

**Background:**

Acid-sensing ion channels (ASICs) are cation channels which were activated by extracellular acidosis and involved in various physiological and pathological processes in the nervous system. Inflammasome is a key component of the innate immune response in host against harmful and irritable stimuli. As the first discovered molecular platform, NLRP1 (nucleotide-binding oligomerization domain (NOD)-like receptor protein 1) inflammasome is expressed in neurons and implicated in many nervous system diseases such as brain injury, nociception and epilepsy. However, little is known about the effect of ASICs on NLRP1 inflammasome activation under acidosis.

**Methods:**

The expression of inflammasome complex protein (NLRP1, ASC (apoptosis-associated speck-like protein containing a caspase-activating recruitment domain) and caspase-1), inflammatory cytokines (IL-1β and IL-18), and apoptosis-related protein (Bax, Bcl-2, and activated caspase-3) was detected by Western blot. Large-conductance Ca^2+^ and voltage-activated K^+^ (BK) channel currents were recorded by whole-cell patch-clamp technology. Measurement of [K^+^]_*i*_ was performed by fluorescent ion imaging system. Co-expression of ASICs and BK channels was determined by dual immunofluorescence. Cell viability was assessed by MTT and LDH kit.

**Results:**

ASICs and BK channels were co-expressed in primary cultured cortical neurons. Extracellular acidosis increased the expression of NLRP1, ASC, caspase-1, IL-1β, and IL-18. Further mechanistic studies revealed that acidosis-induced ASIC1a activation results in the increase of BK channel currents, with the subsequent K^+^ efflux and a low concentration of intracellular K^+^, which activated NLRP1 inflammasome. Furthermore, these effects of acidosis could be blocked by specific ASIC1a inhibitor PcTX1 and BK channel inhibitor IbTX. The data also demonstrated neutralization of NLRP1-protected cortical neurons against injury induced by extracellular acidosis.

**Conclusions:**

Our data showed that NLRP1 inflammasome could be activated by extracellular acidosis though ASIC-BK channel K^+^ signal pathway and was involved in extracellular acidosis-induced cortical neuronal injury.

**Electronic supplementary material:**

The online version of this article (doi:10.1186/s12974-015-0465-7) contains supplementary material, which is available to authorized users.

## Background

Inflammasomes are multi-protein complexes that regulate the activity of caspase-1 and promote the maturation of inflammatory cytokines IL-1β and IL-18, which belong to the family of IL-1 cytokines and have been shown to play a detrimental role after central nervous system (CNS) injury [1]. To date, many inflammasomes have been well characterized such as NLRP1 (nucleotide-binding oligomerization domain (NOD)-like receptor protein 1), NLRP2, NLRP3, NLRC4 (CARD domain-containing protein 4, also called IPAF (ICE-pro tease activating factor)) inflammasome, and AIM2 (absent in melanoma 2) inflammasome [2–6]. The NLRP1 inflammasome is the first to be discovered and composed of NLRP1, an adaptor known as apoptosis-associated speck-like protein containing a caspase-activating recruitment domain (ASC), and caspase-1 [2]. Previous study has shown that NLRP1 neutralization by anti-NLRP1 antibody reduced the inflammatory response and infarct size after common carotid artery thrombosis (CCAT) [7]. Similarly, inhibition of NLRP1 inflammasome by ASC neutralization decreased lesion volume and improved functional outcomes after spinal cord injury (SCI) or traumatic brain injury (TBI) [8, 9]. Furthermore, NLRP1 inflammasome was also implicated in the processes of Alzheimer’s disease (AD), nociception, and epilepsy [10–13]. Thus, NLRP1 inflammasome may play an important role in nervous system diseases.

Proton is the smallest ion but an important physiological indicator of internal environment homeostasis. In the nervous system, protons modulate synaptic transmission, neuronal plasticity, and membrane excitability [14]. However, over-accumulation of protons in the extracellular medium (extracellular acidosis) could result in disturbance of acid–base balance and lead to neuronal damage. Acidosis has been considered as a common feature of many neuronal diseases such as traumatic brain injury, ischemic stroke, epileptic seizure, and neurodegenerative diseases [15–22]. Recently, Jancic and colleagues found that low extracellular pH stimulated the production of IL-1β in human monocytes [23]. Extracellular and intracellular acidosis also activated NLRP3 inflammasome in human macrophages [24]. However, it is unclear whether acidosis influences the activity of NLRP1 inflammasome in neurons.

ASICs are cation channels which belong to the degenerin/epithelial Na+ channel (DEG/ENaC) superfamily and activated by extracellular protons [25]. To date, six ASIC subunit proteins, encoded by four genes, have been identified including ASIC1a, ASIC1b, ASIC2a, ASIC2b, ASIC3, and ASIC4 [26]. They are widely expressed in peripheral sensory neurons and the CNS neurons and play important role in a variety of physiological and pathological processes, such as nociception, mechanosensation, and acidosis-mediated neuronal injury [27, 28]. Many reports showed that ASICs mediate most of the acidosis-associated physiological and pathological functions in the nervous system [29–33]. In addition, ASICs especially ASIC1a may be involved in the activity of NLRP3 under acidic extracellular environment [34]. However, the effects of ASICs on NLRP1 inflammasome activation under acidosis have not been determined.

In the present study, we investigated the effect of extracellular acidosis on the activity of NLRP1 inflammasome and the role of ASICs in the regulation of NLRP1 inflammasome activation under extracellular acidosis. We demonstrated that extracellular acidosis activated NLRP1 inflammasome, and ASICs contribute to the effect of extracellular acidosis on NLRP1 inflammasome activation in primary cultured cortical neurons.

## Methods

### Chemicals

DMEM/F12, fetal bovine serum, and B27 supplement were purchased from Gibco Invitrogen Corporation (Carlsbad, CA, USA). Hoechst 33258, trypsin, iberiotoxin (IbTX), 3-(4, 5-dimethylthiazol-2-yl)-2, 5-diphenyltetrazolium bromide (MTT), and amiloride were obtained from Sigma-Aldrich (St. Louis, MO, USA). The reagent kit for determining lactate dehydrogenase (LDH) was purchased from Nanjing Jiancheng Institute of Biological Engineering (Nanjing, China). Psalmotoxin 1(PcTX1) and primary antibodies of ASIC1, ASIC2, and ASIC3 were purchased from Alomone Labs (Jerusalem, Israel). Additional files showed the specificity of PcTX1 and ASICs antibodies (see Additional files 1 and 2). Potassium-binding benzofuran isophthalate acetoxymethyl ester (PBFI-AM) and pluronic F-127 were obtained from Molecular Probes Inc. (Eugene, OR, USA). Primary antibodies of Bax and Bcl-2 were purchased from Cell Signaling Technology Inc. (San Francisco, CA, USA). Anti-NLRP1 and anti-BK antibody were purchased from Abcam (San Francisco, CA, USA). Primary antibodies of caspase-1, activated-caspase-3, ASC, IL-1β, and IL-18 were purchased from Santa Cruz Biotechnology (Santa Cruz, CA, USA). Horseradish peroxidase-conjugated secondary antibodies were purchased from Santa Cruz Biotechnology (Santa Cruz, CA, USA). Other general agents were commercially available.

### Primary cortical neuron culture

Neonatal Sprague–Dawley (SD) rats (0–24 h) were obtained from the Experimental Animal Center of Anhui Medical University. The University Animal Welfare Committee approved the used animal protocol. Primary rat cortical neurons were isolated and cultured as described in our previous study [35]. Briefly, the cortex of newborn SD rats were dissected and rinsed in ice-cold Dulbecco’s phosphate-buffered saline (PBS). The dissected tissues were treated with 0.125 % trypsin in Hanks’ balanced salt solution for 25 min at 37 °C and mechanically dissociated using a fire-polished Pasteur pipette. Cells were collected by centrifugation and resuspended in DMEM/F12 (1:1) with 10 % fetal bovine serum. For whole-cell patch-clamp recording, cells (20,000–40,000) were seeded on poly-D-lysine coated coverslips and kept at 37 °C in 5 % CO_2_ incubator. After 24 h, the culture medium was changed into DMEM medium supplemented with 2 % B27 and the cortical neurons were fed with fresh medium twice weekly. Microscopically, glial cells were not apparent by employing this protocol. The neurons were maintained for 7–10 days in primary culture until used for whole-cell patch-clamp recording.

### Western blot

After being washed twice with ice-cold PBS, cells were lysed on ice in extraction buffer containing 50 mM Tris-base (pH 7.4), 100 mM NaCl, 1 % NP-40, 10 mM EDTA, 20 mM NaF, 1 mM PMSF, 3 mM Na_3_VO_4_, and protease inhibitors. The homogenates were centrifuged at 12,000 *g* for 15 min at 4 °C. The supernatant was separated and stored at −80 °C until use. Protein concentration was determined using the BCA protein assay kit (Pierce Biotechnology, Inc., Rockford, IL, USA). Protein samples (30 μg) were separated by 10 % SDS-polyacrylamide gel and then transferred to nitrocellulose membranes. After blocking with 5 % nonfat milk in Tris-buffered saline containing 0.1 % Tween-20 (TBST) for 1 h at room temperature, transferred membranes were incubated overnight at 4 °C with different primary antibodies (anti-NLRP1 and anti-activated caspase-3 1:800 dilution; anti-Bax and anti-Bcl-2 1:500 dilution; anti-caspase-1, anti-ASC, anti-IL-1β, and anti-IL-18 1:200 dilution). Following three washes with TBST, membranes were then incubated with horseradish peroxidase-conjugated secondary antibodies (1:10 000) in TBST with 1 % nonfat milk for 1 h at room temperature. After repeated washes, membranes were reacted with enhanced chemiluminescence reagents (Amersham Pharmacia Biotech, Inc., Piscataway, NJ, USA) for 5 min and visualized with X-ray films (Kodak X-Omat, Rochester, NY, USA). The films were scanned, and the optical density of the bands was determined using Optiquant software (Packard Instrument). Results are expressed as percentage of control signals (% control) in each blot to correct for variations between blots.

### Measurement of intracellular K^+^ concentration

Measurement of [K^+^]_*i*_ was performed as described by Kozoriz et al. [36] with minor modifications. In brief, the cells were washed three times with artificial cerebrospinal fluid (ACSF) containing (in millimolar) the following: 140 NaCl, 5 KCl, 1 MgCl_2_, 2 CaCl_2_, 10 glucose, and 10 HEPES (pH 7.3) then loaded with 5 μM PBFI and 0.05 % pluronic F-127 in ACSF for 1 h at room temperature. The cells were then placed in fluorophore-free medium for 30 min and then mounted on a chamber positioned on the movable stage of an inverted microscope (TE2000, Japan), which was equipped with a ion imaging system (PTI, USA). The cells were superfused by ACSF at a rate of 2 ml/min for 10 min. Fluorescence was excited at wavelengths of 340 and 380 nm at 1-s interval by a monochromator (PTI K-178-S), and the emission was imaged at 510 nm with a video camera (CoolSNAP HQ2, ROPPER, USA) through fluor oil-immersion lens (Nikon) and a wideband emission filter. F340/F380 fluorescence ratio was recorded and analyzed by MetaFluor version 6.3 software. Results are expressed as percentage of control signals (% control).

### Whole-cell patch-clamp recording

The procedure for whole-cell patch-clamp recording was executed as that described in our previous reports with minor modification [37, 38]. The bath solution for recording BK channel currents was composed of (in millimolar) the following: 144 NaCl, 6 KCl, 1.2 MgCl_2_, 2 CaCl_2_, 10 HEPES, 10 D-glucose, and 5 4-AP, pH adjusted to 7.4 with NaOH. Glass pipettes were used with a resistance of 2–4 MΩ when filled with the following solution (in millimolar): 110 K-glutamine, 20 KCl, 3 Na_2_ATP, 0.1 EGTA, 3 MgCl_2_, 10 HEPES, and 10 D-glucose, pH adjusted to 7.2 with KOH. After establishing a whole-cell configuration, the adjustment of capacitance compensation and series resistance compensation was done before recording. The current signals were acquired at a sampling rate of 10 kHz and filtered at 3 kHz. Whole-cell patch-clamp recordings were carried out using an EPC-10 amplifier (HEKA, Lambrecht, Germany) driven by Pulse/PulseFit software (HEKA, Southboro, Germany). Drug actions were measured only after steady-state conditions reached, which were judged by the amplitudes and time courses of currents remaining constant. All the recordings were made at room temperature (20–22 °C). All experiments were repeated three times using different batches of cells and at least the 3–4 dishes with cells were used for recording in different batches of cells.

### Dual immunofluorescence experiments

Cortical neurons were fixed with 4 % paraformaldehyde for 30 min and rinsed three times (10 min each) with PBS, permeabilized with 0.3 % Triton X-100 for 30 min, and blocked with 3 % bovine serum albumin (BSA)-PBS for 30 min. The neurons were incubated with 1:50 anti-ASIC antibodies and anti-BK antibodies overnight at 4 °C. Then, the cells were rinsed three times (10 min each) with PBS, incubated with 1:100 FITC-conjugated goat anti-rabbit IgG and TRITC-conjugated goat anti-mouse IgG for 1 h at room temperature. After being washed with PBS, cells were incubated with Hoechst 33258 for 15 min. Finally, cells were mounted with coverslips and visualized with confocal microscopy.

### Assessments of cell viability

After various treatments, cell viability was measured by using the MTT assay, which was based on the conversion of MTT to formazan crystals by mitochondrial dehydrogenases. Cell cultures were incubated with MTT solution (5 mg/ml) for 4 h at 37 °C. Then, the medium was discarded and dimethyl sulphoxide (DMSO) was added to solubilize the reaction product formazan by shaking for 15 min. Absorbance at 492 nm was measured with a microplate reader (ELx800, Bio-Tek, Winooski, VT, USA). Cell viability of vehicle group that was not exposed to acidosis was defined as 100 %. Cell viability was expressed as a percentage of the value in control group. To confirm the cell death, the amount of LDH released to the medium and total LDH were determined after 24 h of acidosis. The assay of LDH activity was performed according to the protocols of LDH kit. Briefly, an aliquot of the culture supernatants (extracellular) or cell dissociation solution (intracellular) was mixed with nicotinamide adenine dinucleotide (NAD) and lactate solution. Colorimetric absorbance was measured at 490 nm with a microplate reader. Total LDH activity was calculated by adding the values for extracellular and intracellular LDH that were measured in live cells treated with 1 % Triton X-100. The ratio of released LDH (extracellular) vs total LDH (extracellular + intracellular) was calculated and expressed as a percentage of total LDH.

### Statistical analysis

Data from experiments were analyzed with the statistical program SPSS (SPSS, Chicago, IL, USA). A two-sided Student’s *t* test with paired comparisons was used to evaluate differences in electrophysiological data. For other data, comparison between two groups was evaluated by a two-sided and unpaired Student’s *t* test. Data are expressed as means ± SEM. *p* < 0.05 were considered statistically significant.

## Results

### Extracellular acidosis activates NLRP1 inflammasome in cortical neurons

**Fig. 1:**
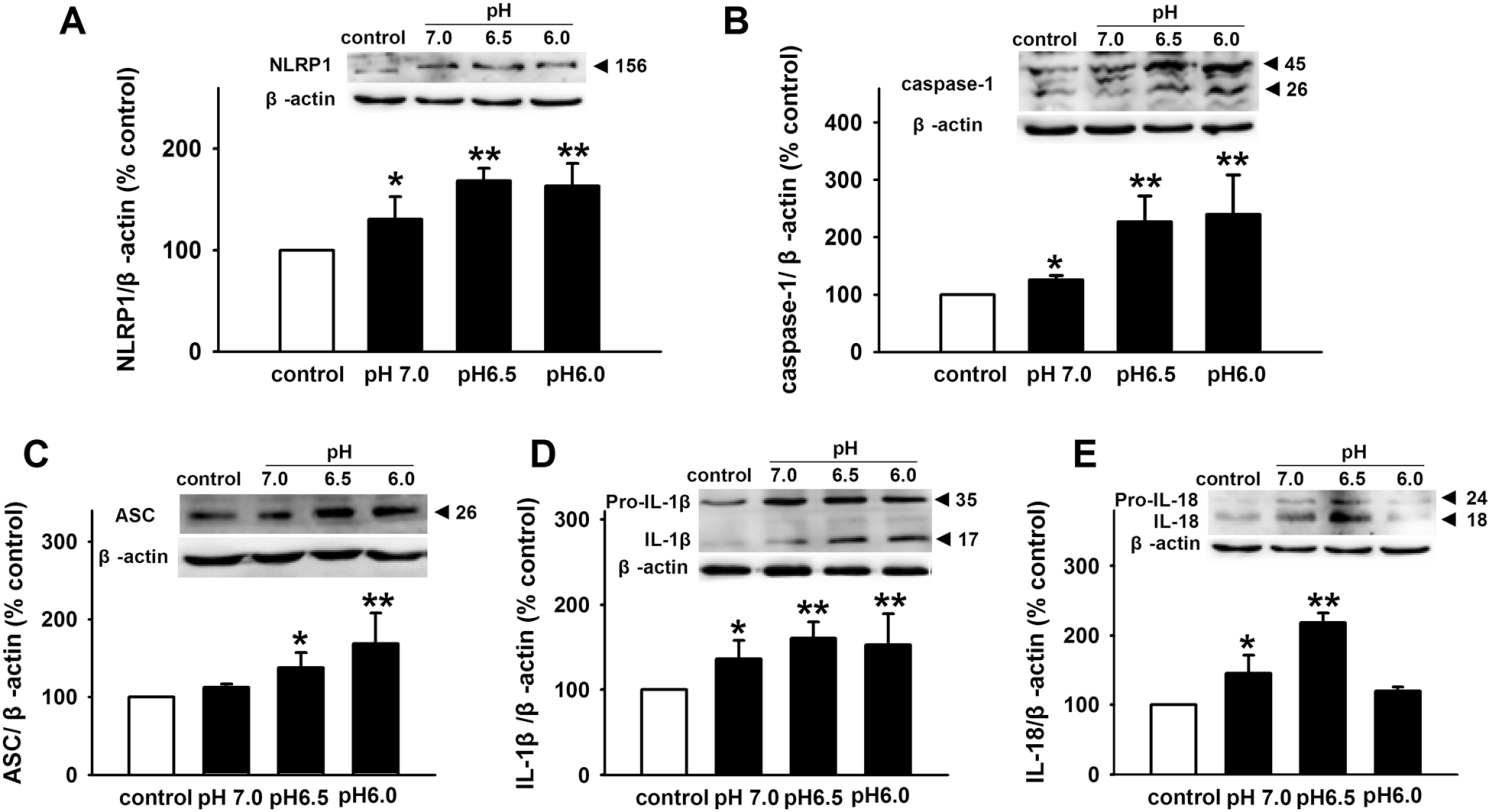
Extracellular acidosis activates NLRP1 inflammasome in primary cultured cortical neurons. Representative immunoreactive bands and statistical results showing low pH extracellular medium increases the expression of NLRP1 (**a**), caspase-1 (**b**), ASC (**c**), IL-1β (**d**), and IL-18 (**e**). Data are expressed as means ± SEM. *n* = 6, *p < 0.05 and **p < 0.01 vs control

In order to observe the effect of extracellular acidosis on NLRP1 inflammasome activation, cortical neurons were pretreated with extracellular medium at different pH (pH 7.0, pH 6.5, and pH 6.0) for 4 h, followed by reperfusion for 24 h. Then, the expression of NLRP1, caspase-1, ASC, IL-1β, and IL-18 was detected by Western blot. As shown in Fig. 1, compared with the control group, extracellular acidosis significantly increased the expression of NLRP1, caspase-1, ASC, IL-1β, and IL-18. Except IL-18, the effects of acidosis are pH dependent. Moreover, these effects are most significant at pH 6.5. So, we selected pH 6.5 for acid stimuli in subsequent experiments. These results suggest that extracellular acidosis activates NLRP1 inflammasome in primary cultured cortical neurons.

### Extracellular acidosis reduces [K^+^]_*i*_ by BK channels

**Fig. 2:**
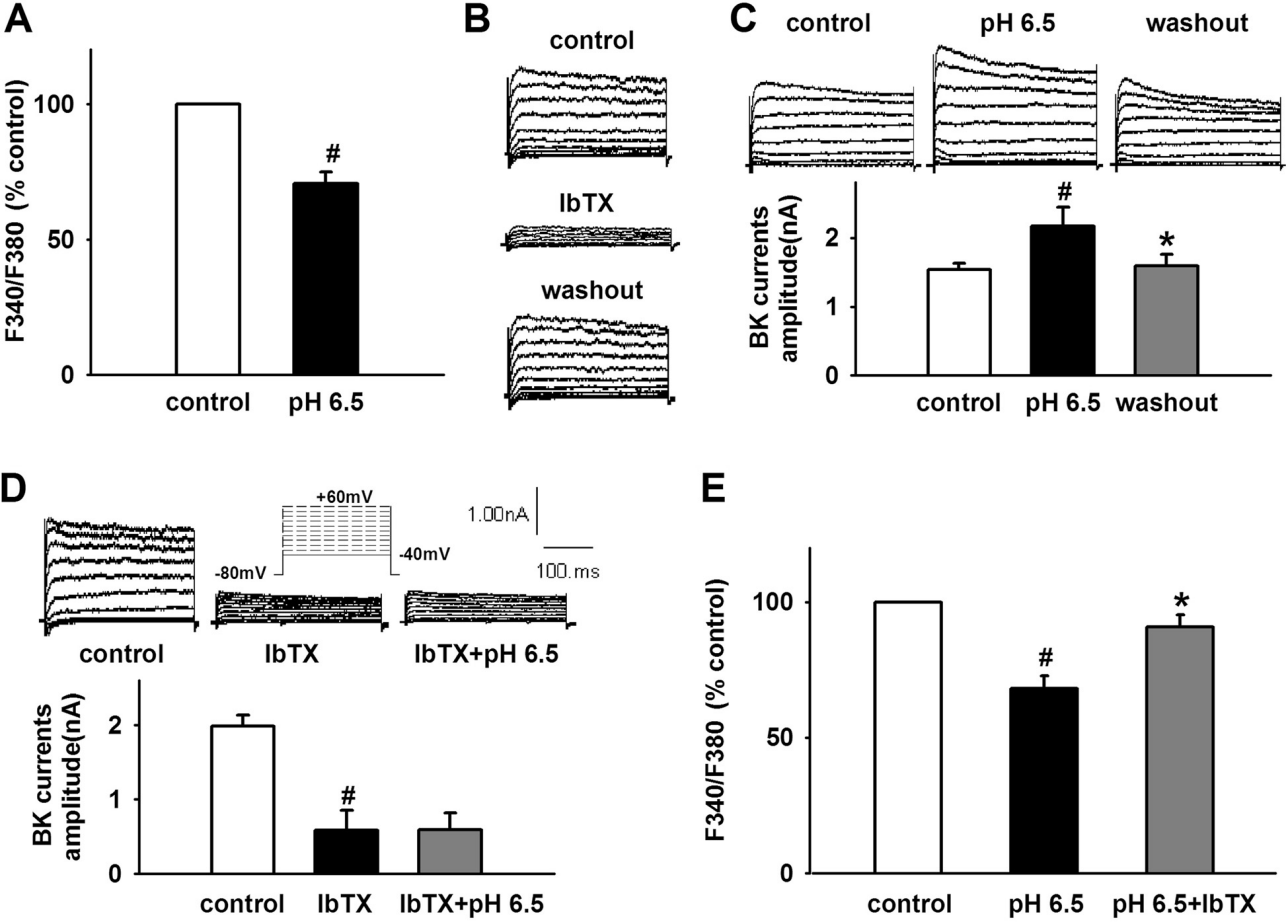
Extracellular acidosis reduces [K^+^]_*i*_ by BK channel in cortical neurons. **a** Statistical results showing low pH extracellular medium reduced [K]_*i*_. **b** Representative traces showing recorded potassium currents were markedly inhibited by specific BK channel inhibitor IbTX. **c** Representative traces and statistical results showing low pH extracellular medium increased BK currents. **d** Representative traces and statistical results showing low pH extracellular medium failed to increase BK currents in the presence of IbTX. **e** Statistical results showing IbTX attenuated acidosis-induced decrease in [K]_*i*_. Data are expressed as means ± SEM. *n* = 6, ^#^ *p* < 0.05 vs control and *p < 0.05 vs pH 6.5

Recently, the role of K^+^ in NLRP1 inflammasome activation is well interpreted [34]. To determine whether K^+^ is involved in the activity of NLRP1 inflammasome induced by extracellular acidosis, we observed the change of [K^+^]_*i*_ under acidosis. The results showed that pH 6.5 extracellular medium significantly reduces [K^+^]_*i*_ to 70.56 ± 4.46 % in cortical neurons (Fig. 2a). BK channels, which are activated by Ca^2+^ rise and depolarization, influenced cell excitability and neurotransmitter release in the CNS. In addition, BK channels are also important for K^+^ transport[39]. To investigate whether BK channels contribute to the decrease of [K^+^]_*i*_ induced by extracellular acidosis, we recorded BK channel currents under acid stimuli by whole-cell patch-clamp technology. As shown in Fig. 2b, BK channel currents were elicited as described in our previous reports [37] by applying 11 depolarizing pulses from −40 to +60 mV for 500 ms with a 10-mV increment from a holding potential of −80 mV. To confirm that the recorded currents were mediated by BK channels, specific BK channel inhibitor IbTX was used. The results showed that IbTX (200 nM) markedly decreased the peak amplitude of recorded currents by 74.26 ± 5.36 % (*n* = 6, *p* < 0.05), suggesting that recorded currents were carried by BK channels. Furthermore, acidic extracellular medium (pH 6.5) significantly increased BK currents from 1.54 ± 0.09 to 2.16 ± 0.28 nA in cortical neurons. After washout, BK currents returned to the control level (Fig. 2c). While pH 6.5 failed to increase BK currents in the presence of IbTX (200 nM) (Fig. 2d), indicating that the current potentiation induced by pH 6.5 is sensitive to IbTX. Similarly, 200 nM of IbTX significantly attenuated the effects of extracellular acidosis on [K]_*i*_ in cortical neurons (Fig. 2e). These data suggested that BK channels contributed to the effect of extracellular acidosis on [K]_*i*_ in cortical neurons.

### ASICs interact with BK channels and mediate the decrease of [K^+^]_*i*_ induced by extracellular acidosis

**Fig. 3:**
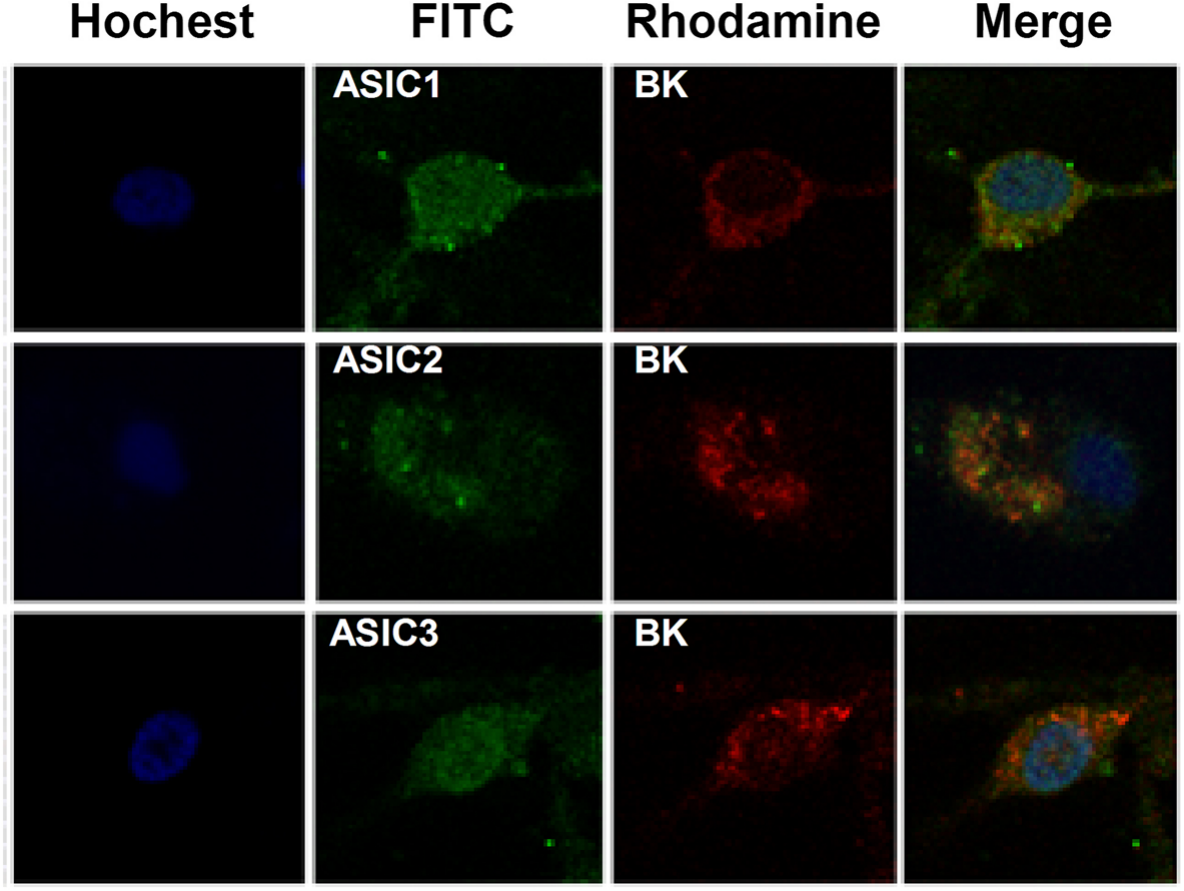
ASICs and BK channels are co-expressed in cortical neurons. Detection of the expression of ASICs and BK channels in cortical neurons by double-staining immunofluorescence (original magnification ×400). Nuclei were counterstained with Hoechst33258 (*blue*). ASICs and BK channels were labeled with FITC (*green*) and TRITC (*red*). There is a co-expression between ASIC1, ASIC2, ASIC3, and BK channels

**Fig. 4:**
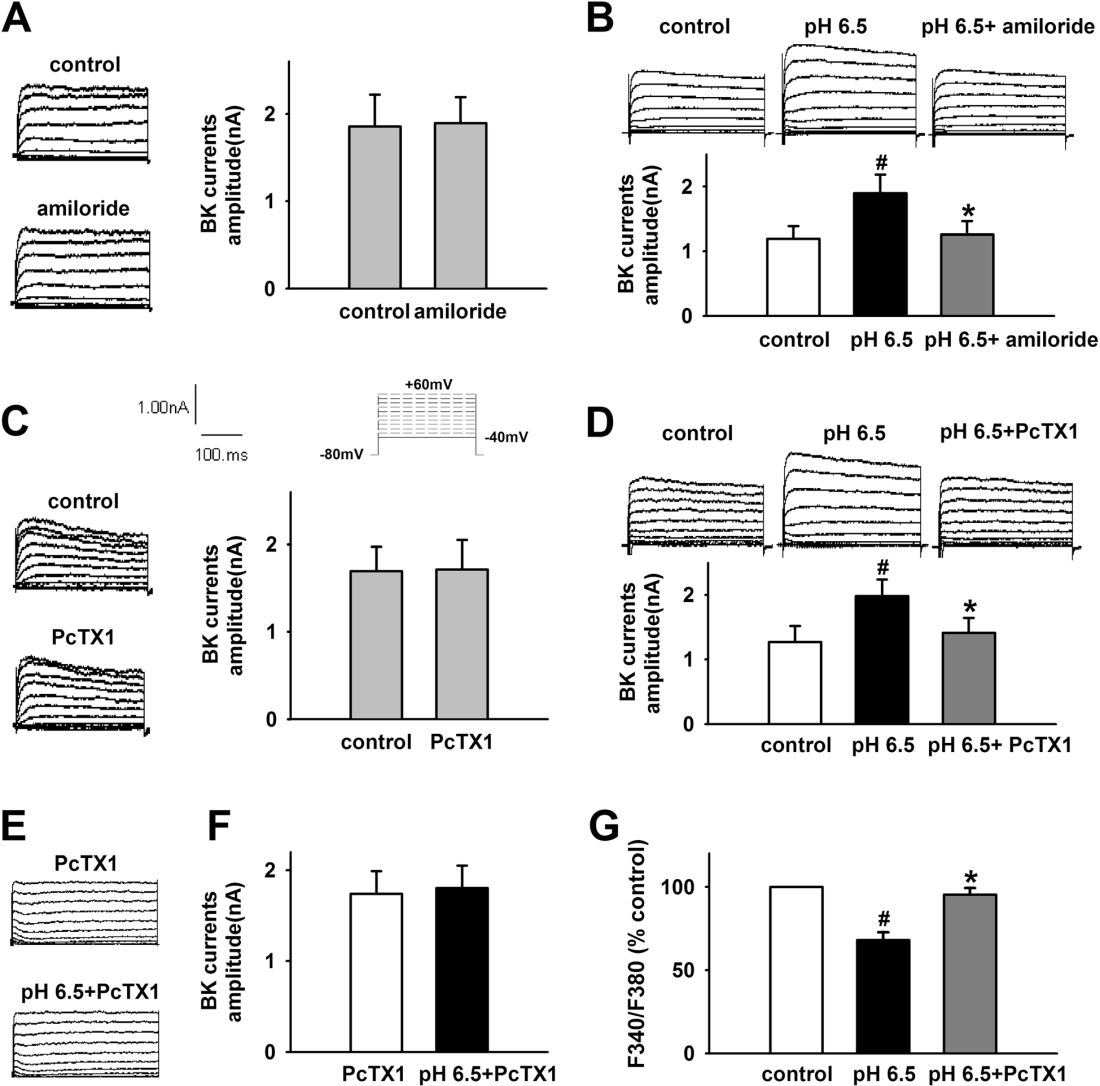
ASICs mediate the increase of BK currents and the decrease of [K^+^]_*i*_ induced by extracellular acidosis. **a** Representative traces and statistical results showing amiloride did not influence BK currents at neutral pH. **b** Representative traces and statistical results showing amiloride inhibited acidosis-induced increase of BK currents. **c** Representative traces and statistical results showing PcTX1 did not influence BK currents at neutral pH. **d** Representative traces and statistical results showing PcTX1 inhibited acidosis-induced increase of BK currents. **e**, **f** Representative traces and statistical results showing acidosis failed to increase BK currents after pretreatment with PcTX1. **g** PcTX1 attenuated acidosis-induced decrease in [K^+^]_*i*_. Data are expressed as means ± SEM. *n* = 6, ^#^ *p* < 0.05 vs control and *p < 0.05 vs pH 6.5

Previous study demonstrated that ASICs interact with BK channels and inhibit BK channels in HEAK 293 cells expressing the two channels. Extracellular acidosis activated ASICs and attenuated inhibition effects resulting in the increase of BK channel currents [40]. To determine the role of ASICs in the effect of extracellular acidosis on BK channel and [K^+^]_*i*_ in cortical neurons, we first observe whether there is a co-expression between ASICs and BK channels. The results showed that ASIC1, ASIC2, and ASIC3 co-express with BK channel in primary culture cortical neurons (Fig. 3). Then, we further investigate the effect of ASICs on BK channels under acid stimuli. Similar to above results, pH 6.5 extracellular medium significantly increased BK currents from 1.19 ± 0.19 to 1.89 ± 0.28 nA (Fig. 4b). Amiloride (100 nM), a non-selective ASIC inhibitor, did not influence BK currents at neutral pH (Fig. 4a). However, it significantly inhibits the potentiation of BK currents under acidosis (Fig. 4b). Similarly, PcTX1 (10 nM), a specific ASIC1a inhibitor, did not influence BK currents at neutral pH either (Fig. 4c). However, it blocked the effects of acidosis on BK currents (Fig. 4d), indicating that ASIC1a is responsible for BK current increase induced by acidosis. To further demonstrate the effects, we pretreated cortical neurons with PcTX1 (10 nM), and then the BK channel currents were recorded under acid stimuli. The results showed that extracellular acidosis failed to increase BK channel currents (Fig. 4e, f). That further confirmed that ASIC1a-mediated BK currents increase induced by acidosis. As expected, PcTX1 (10 nM) also attenuated the effects of extracellular acidosis on [K]_*i*_ (Fig. 4g)_._ All these results suggested that ASIC1a contributes the to the effect of extracellular acidosis on BK channels and [K^+^]_*i*_

### Blockage of ASIC1a and BK channels attenuates NLRP1 inflammasome activation induced by extracellular acidosis

**Fig. 5:**
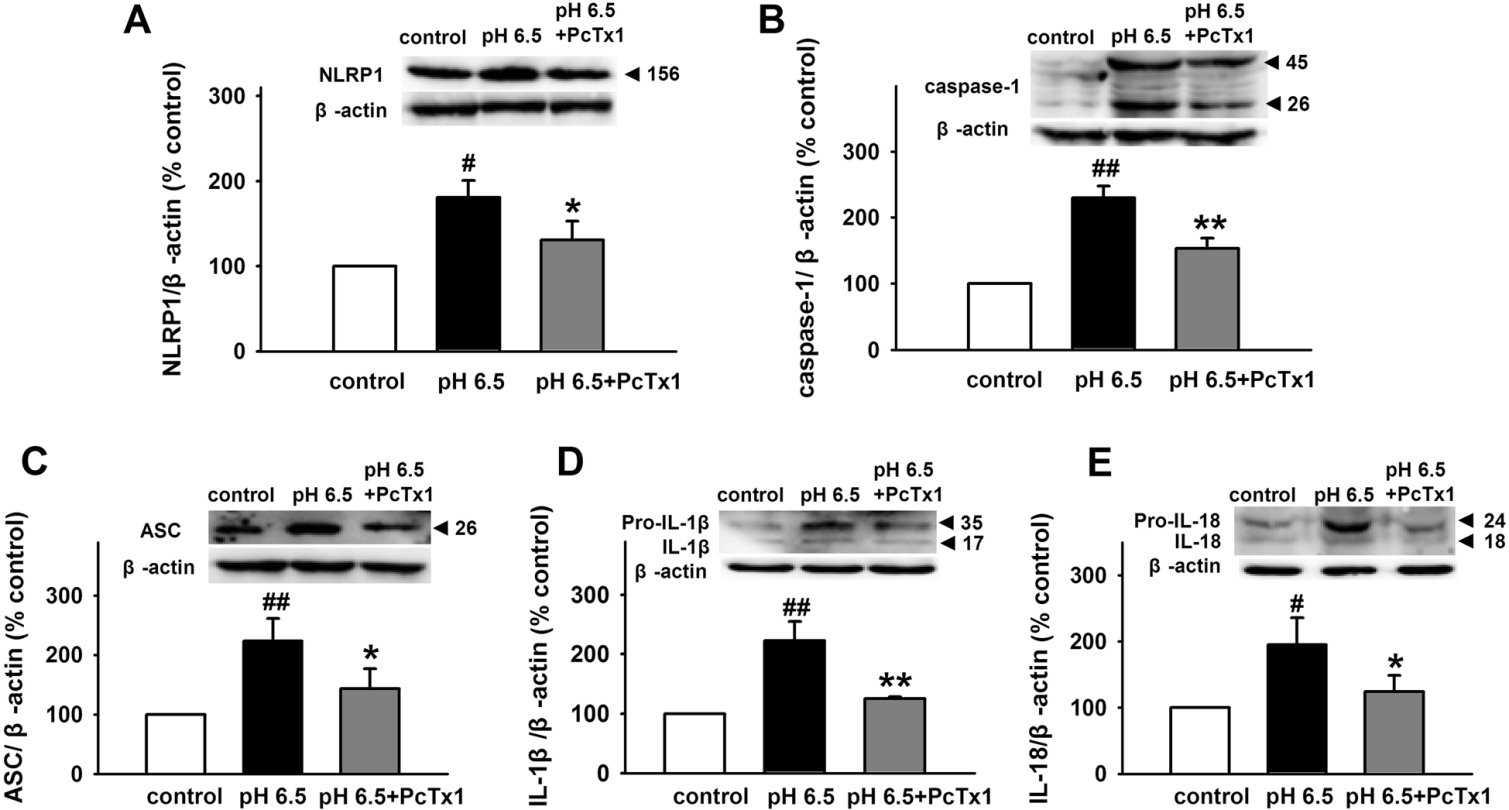
Blockage of ASICs inhibits NLRP1 inflammasome activation induced by extracellular acidosis**.** Representative immunoreactive bands and statistical results showing PcTX1 attenuated acidosis-induced increase in the expression of NLRP1 (**a**), caspase-1 (**b**), ASC (**c**), IL-1β (**d**), and IL-18 (**e**). Data are expressed as means ± SEM. *n* = 6, ^#^ *p* < 0.05 and ^##^p < 0.01 vs control, *p < 0.05 and **p < 0.01 vs pH 6.5

**Fig. 6:**
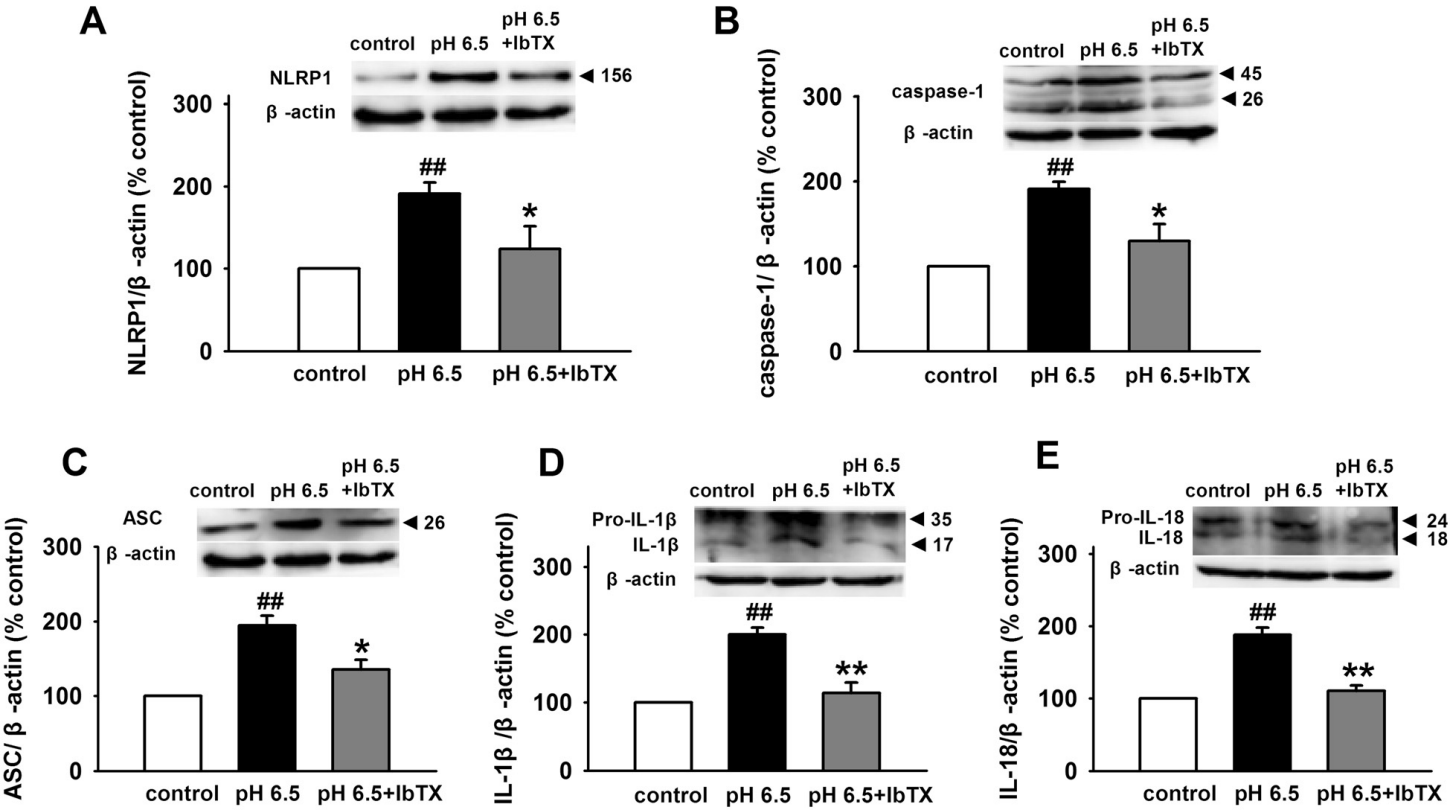
Blockage of BK channel inhibits NLRP1 inflammasome activation induced by extracellular acidosis. Representative immunoreactive bands and statistical results showing IbTX attenuated acidosis-induced increase in the expression of NLRP1 (**a**), caspase-1 (**b**), ASC (**c**), IL-1β (**d**), and IL-18 (**e**). Data are expressed as means ± SEM. n = 6, ^#^ *p* < 0.05 and ^##^ *p* < 0.01 vs control, *p < 0.05 and **p < 0.01 vs pH 6.5

Above data have demonstrated that extracellular acidosis activates ASIC1a and results in the increase of BK channel currents and the decrease of [K]_*i*_, which may be responsible for NLRP1 inflammasome activation induced by extracellular acidosis. To further testify the hypothesis, specific ASIC1a inhibitor PcTX1 and BK channels inhibitor IbTX were used in our experiment. As shown in Fig. 5, compared to control group, extracellular acidosis significantly increased the expression of NLRP1, caspase-1, ASC, IL-1β, and IL-18 and activated NLRP1 inflammasome. However, PcTX1 (10 nM) significantly attenuated the effects (Fig. 5). Similarly, the effects were also blocked by IbTX (200 nM) (Fig. 6). Taken together, these results suggested ASIC-BK channel K^+^ signal mediates the effect of extracellular acidosis on NLRP1 inflammasome activation.

### Neutralization of NLRP1 protects against acidosis-induced neuronal injury

**Fig. 7:**
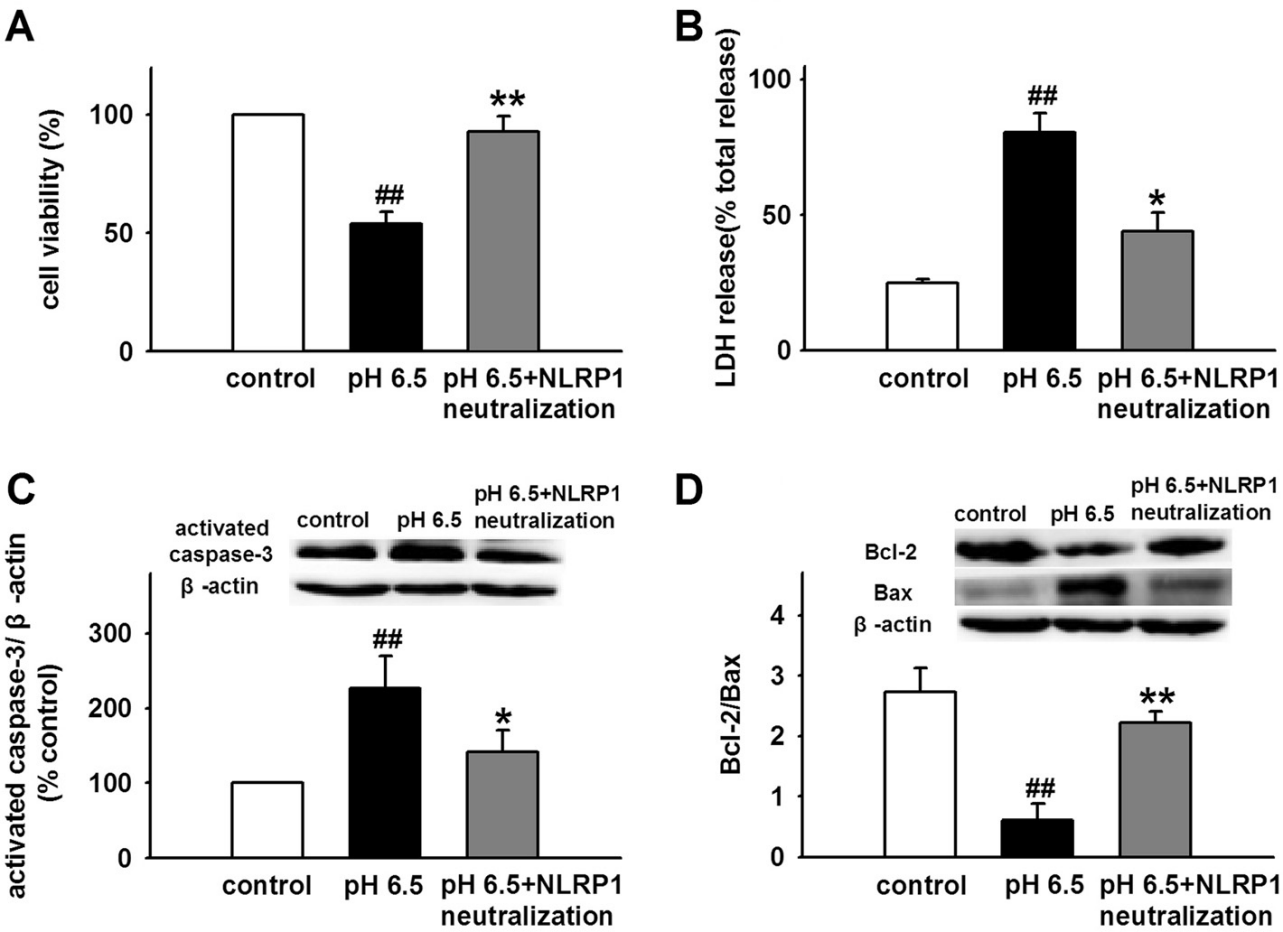
Neutralization of NLRP1 protects against neuronal injury induced by extracellular acidosis. **a**, **b** Statistical results showing NLRP1 neutralization attenuated acidosis-induced decrease in cell viability and increase in LDH release. **c**, **d** Representative immunoreactive bands and statistical results showing NLRP1 neutralization attenuated acidosis-induced decrease in ratio of Bcl-2/Bax and increase in expression of activated caspase-3. Data are expressed as means ± SEM. *n* = 6, ^##^ *p* < 0.01 vs control, *p < 0.05 and **p < 0.01 vs pH 6.5

Acidosis-induced cell injury or death is well demonstrated in cultured hippocampal, cortical, and cerebellar granule neurons [29, 41, 42]. The Ca^2+^-permeable ASIC1a-induced calcium overload is a critical element in the neuronal damaging cascade [43]. Our results have shown that ASIC1a mediates the activity of NLRP1 inflammasome induced by extracellular acidosis. To determine whether NLRP1 inflammasome is involved in acidosis-induced neuronal injury, we blocked the activity of NLRP1 with anti-NLRP1 antibody (10 μg/ml) and measured cell viability, LDH release, and the expression of Bcl-2/Bax and caspase-3 under acidosis. We found that extracellular acidosis (pH 6.5) reduced cell viability and increased LDH release; while NLRP1 neutralization significantly attenuated the effects (Fig. 7a, b). Similarly, extracellular acidosis (PH 6.5) reduced the ratio of Bcl-2/Bax and increased the expression of activated caspase-3, and NLRP1 neutralization also blocked the effects of acidosis (Fig. 7c, d), indicating NLRP1 inflammasome is dominantly involved in neuronal injury induced by extracellular acidosis.

## Discussion

In the current study, we demonstrated that NLRP1 inflammasome is activated under acidosis, and ASIC-BK channel K^+^ signal mediates NLRP1 inflammasome activation induced by extracellular acidosis, which is accountable for acidosis-induced cortical neuronal injury.

Inflammation is an innate immune response to infection and tissue damage, and it is designed to limit harm to the host. Inflammasome, a key component of the innate immune response, plays a critical role in the pathology after injury to the CNS. As the first identified inflammasome, NLRP1 inflammasome is expressed in neurons and involved in many nervous system diseases such as thromboembolic stroke, SCI, TBI, AD, nociception, and epilepsy [7–10, 12, 13]. As we know, inflammation is always accompanied by local tissue acidification. However, the effect of an acidic microenvironment on the activity of NLRP1 inflammasome is unclear. To investigate the effects of acidosis on NLRP1 inflammasome activation, the expression of inflammasome complex was tested under an acidic condition. We found that extracellular acidosis increased the expression of NLRP1, ASC, and caspase-1 in a pH-dependent manner. Also, extracellular acidosis stimulated the production of inflammatory cytokines IL-1β and IL-18, indicating that extracellular acidosis activates NLRP1 inflammasome in cortical neurons.

Although NLRP1 inflammasome is the first to be characterized, the regulation of its activation is less well known. Initial studies showed that the spontaneous activation of NLRP1 inflammasome occurs in a cellular lysate [2]. Recent studies showed that several stimuli contribute to the activity of NLRP1 inflammasome, including *Bacillus anthracis* lethal toxin, *Toxoplasma gondii*, muramyl dipeptide, and host intracellular ATP depletion [44–48]. Recently, the role of K^+^ in the activity of inflammasome is well documented. Low concentrations of intracellular K^+^ (below 90 mM) activate NLRP1 inflammasome in immune cells [49]. Also, valinomycin-triggered K^+^ efflux activates caspase-1 and increases IL-1β secretion in cultured spinal cord neurons [8]. Similarly, experimental depletion of serum K^+^ from culture medium greatly increases NLRP1 expression in rat cerebellar granule neurons [50]. However, Silverman et al. found that high concentration of extracellular K^+^ ([K^+^]_*o*_) activated NLRP1 inflammasome through pannexin-1 channel in neurons and astrocytes [51]. The mechanism may be related to a positive feed-forward loop: the efflux of K^+^ through the open P2X4 channel leads to the increase in [K^+^]_*o*_ and triggers the activity of pannexin-1 channel and then further activates P2X4 and P2X7 receptor channel [52]. Thus, [K^+^]_*i*_ may be a critical element in the activity of NLRP1 inflammasome. To determine the role of K^+^ in NLRP1 inflammasome activation under acidosis, [K^+^]_*i*_. was detected in cortical neurons. We found that extracellular acidosis decreased [K^+^]_*i*_ and increased BK channel currents. Interestingly, IbTX, a specific inhibitor of BK channel, attenuated the effect of acidosis on BK currents and [K^+^]_*i*_, indicating that acidosis-induced NLRP1 inflammasome activation results from the decrease of [K^+^]_*i*_ though BK channel in cortical neurons. Meanwhile, we also noticed that pH 6.0 extracellular medium increases BK currents and reduces [K^+^]_*i*_ (see Additional file 3) but does not increase IL-18 expression. Now, it is unclear what is responsible for the phenomenon. Maybe the reason is that serious acidosis (pH 6.0) elicited a heat-shock response. Previous study has confirmed that heat-shock response can downregulate IL-18 expression [53]. However, further efforts will be made to clarify the precise mechanism in future research.

BK channels, which are gated by Ca^2+^ and voltage, contribute to action potential repolarization in neurons and play an important role in regulating neurotransmitter release [37, 39]. Outward K^+^ currents through BK channels repolarize the cell and reduce excitability. Furthermore, mitoBK channels are important for the K^+^ transport [39]. Recent study has shown that ASIC1a interacts with and inhibits BK channels in HEAK293 cells with co-expression of these two channels. In rest state, ASIC1a inhibited the activity of BK channel. However, extracellular acidosis could activate ASIC1a and relieve the inhibitory, followed by the increase in BK channel currents [40]. Similarly, Petroff et al. found that in cortical neurons of the ASIC knockout mice, the amplitude of BK channel currents was larger and action potentials were narrow and exhibited increased after-hyperpolarization [54]. Therefore, we proposed that extracellular acidosis-induced ASIC activation reduces the inhibitory effect and increases BK channel currents, which contribute to the lower [K^+^]_*i*_ and the activity of NLRP1 inflammasome in cortical neurons. To test this hypothesis, first, we investigated the co-expression of ASICs and BK channels and found that various ASIC subtypes and BK channels are co-expressed in primary cultured cortical neurons. Then, we found that extracellular acidosis increased BK channel currents and amiloride, a non-selective ASIC inhibitor, reduced the effect. Similarly, PcTX1, a specific ASIC1a inhibitor, also attenuated the effect of acidosis on BK currents. Interestingly, after pretreatment of cortical neurons with PcTX1, extracellular acidosis failed to increase BK channel currents, indicating that ASIC1a contributed dominantly to the effect of acidosis on BK channels. PcTX1 also attenuated the decrease of [K^+^]_*i*_ induced by extracellular acidosis, suggesting that ASIC1a mediates the increase of BK channel currents and the decrease of [K^+^]_*i*_ under extracellular acidosis. Finally, we found that blocking ASIC1a with PcTX1 inhibited the activity of NLRP1 inflammasome. Similar effect was also observed after blocking of BK channels with IbTX. Collectively speaking, we demonstrated that ASIC -BK channel K^+^ signal contributes to the activity of NLRP1 inflammasome induced by extracellular acidosis in primary cultured cortical neurons.

Acidosis is a pathological condition in which acid–base balance is disturbed because of over-accumulation of acidity in the body fluid. It is a common phenomenon of many CNS diseases. Before the discovery of acid-sensing ion channels (ASICs),the effects of acidosis on neuronal function were considered as consequences of modulations of ion channels such as voltage-gated calcium channels, *N*-methyl-D-aspartate, and γ-aminobutyric acid (A) receptor channels [14]. However, the conventional view on acidosis-mediated cell injury has been dramatically changed since ASICs were characterized. Calcium overload that resulted from Ca^2+^-permeable ASIC1a activity mediates most of the acidosis-associated pathological processes such as brain ischemia, traumatic brain injury, and neurodegenerative diseases [19, 29, 31, 32]. In addition, Zn^2+^ was also reported to be involved in acidosis-induced neuronal death [55]. It indicates that the disorder of intracellular ion balance may be a critical factor in neuronal death induced by acidosis. Our results have shown that extracellular acidosis altered the concentration of intracellular K^+^ and leaded to lower [K^+^]_*i*_ and NLRP1 inflammasome activation. Thus, lower [K^+^]_*i*_ induced NLRP1 inflammasome activation may be an important element in acidosis-induced neuronal injury. To further confirm the hypothesis, the cell viability and apoptosis were investigated. We found that extracellular acidosis triggered neurons damage, while neutralization of NLRP1 blocked acidosis-induced neuronal death, indicating that NLRP1 inflammasome is critically involved in acidosis-induced neuronal injury.

## Conclusions

The present study demonstrated that extracellular acidosis activates NLRP1 inflammasome through decreasing [K^+^]_*i*_ via ASIC1a-BK channel signal pathway. Neutralization of NLRP1 protected against neuronal injury induced by extracellular acidosis. Therefore, inhibition of NLRP1 inflammasome may offer a new therapy for acidosis-induced neuronal damage in various acidosis-associated nervous system diseases.

## Additional files

Additional file 1: Figure S1. Identification of specificity of ASICs antibodies.Detection of ASIC protein in cortical neurons by double-staining immunofluorescence (original magnification ×400). Nuclei were counterstained with Hoechst33258 (blue). ASICs were labeled with FITC (green). ASIC1, ASIC2, and ASIC3 protein cannot be detected after pretreatment with the corresponding antigen (Ag) peptide. (PDF 106 kb) Additional file 2: Figure S2. The effect of PcTX1 on ASIC currents in cortical neurons. Representative traces and statistical results showing PcTX1 (10 nM) markedly decreased the peak amplitude of ASIC current from 815.62 ± 58.64 to 435.33 ± 61.67 pA. After washout, ASIC currents returned to the control level. Data are expressed as means ± SEM. *n* = 6, ^#^ *p* < 0.05 vs control and **p* < 0.05 vs PcTX1. (PDF 99 kb) Additional file 3: Figure S3. The effect of pH 6.0 extracellular medium on BK currents and [K^**+**^]_*i*_ in cortical neurons. Representative traces and statistical results showing pH 6.0 extracellular medium increased BK currents from 1.29 ± 0.1 to 1.99 ± 0.19 nA (A) and reduced [K^+^]_*i*_ to 65.16 ± 5.77 % (B). Data are expressed as means ± SEM. *n* = 6, ^#^ *p* < 0.05 vs control and **p* < 0.05 vs pH 6.0. (PDF 155 kb)

## Abbreviations

ACSF: artificial cerebrospinal fluid
AIM2: absent in melanoma 2
ASC: apoptosis-associated speck-like protein containing a caspase-activating recruitment domain
ASICs: acid-sensing ion channels
BK channels: large-conductance Ca^2+^ and voltage-activated K^+^ channels
CCAT: common carotid artery thrombosis
CNS: central nervous system
DEG/ENaC: degenerin/epithelial Na + channel
DMSO: dimethyl sulphoxide
IbTX: iberiotoxin
IL-1β: interleukin-1β
IL-18: interleukin-18
IPAF: ICE-protease activating factor
LDH: lactate dehydrogenase
MTT: 3-(4, 5-dimethylthiazol-2-yl)-2,5-diphenyltetrazolium bromide
NAD: nicotinamide adenine dinucleotide
NLRC4: CARD domain-containing protein 4
NLRP1: nucleotide-binding oligomerization domain (NOD)-like receptor protein 1
PBFI-AM: potassium-binding benzofuran isophthalate acetoxymethyl ester
PBS: phosphate-buffered saline
PcTX1: psalmotoxin 1
SCI: spinal cord injury
TBI: traumatic brain injury
TBST: Tris-buffered saline containing 0.1 % Tween-20
