# Supplementary material for: Acid-sensing ion channel 1a contributes to the effect of extracellular acidosis on NLRP1 inflammasome activation in cortical neurons
Source: J Neuroinflammation. 2015 Dec 30;12:246. doi: 10.1186/s12974-015-0465-7 (PMC4696203; doi:10.1186/s12974-015-0465-7)
Supplement: Additional file 1: Figure S1. — Identification of specificity of ASICs antibodies.Detection of ASIC protein in cortical neurons by double-staining immunofluorescence (original magnification ×400). Nuclei were counterstained with Hoechst33258 (blue). ASICs were labeled with FITC (green). ASIC1, ASIC2, and ASIC3 protein cannot be detected after pretreatment with the corresponding antigen (Ag) peptide. (PDF 106 kb) [file 12974_2015_465_MOESM1_ESM.pdf]

Additional file 1

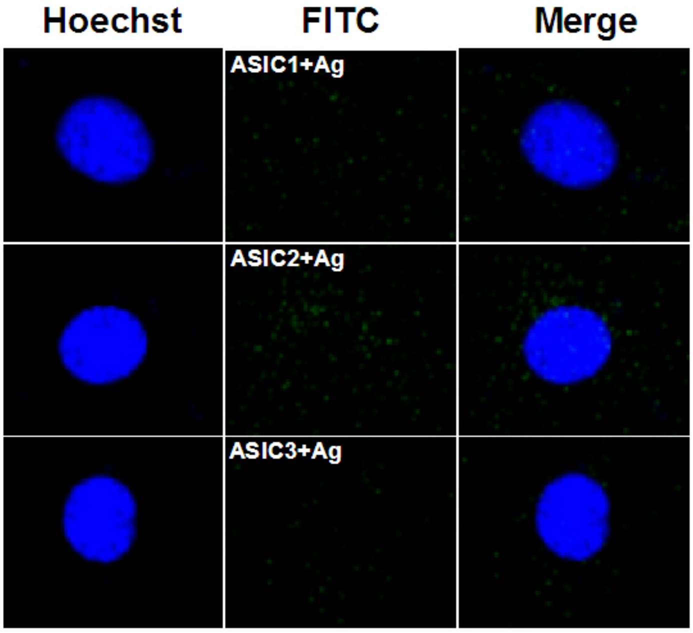

SFig1. Identification of specificity of ASICs antibodies. Detection of ASICs protein in cortical neurons by double-staining immunofluorescence (original magnification×400). Nuclei were counterstained with Hoechst33258 (blue). ASICs were labeled with FITC (green). ASIC1, ASIC2 and ASIC3 protein do not be detected after pretreatment with the corresponding antigen (Ag) peptide
