# Supplementary material for: Acid-sensing ion channel 1a contributes to the effect of extracellular acidosis on NLRP1 inflammasome activation in cortical neurons
Source: J Neuroinflammation. 2015 Dec 30;12:246. doi: 10.1186/s12974-015-0465-7 (PMC4696203; doi:10.1186/s12974-015-0465-7)
Supplement: Additional file 2: Figure S2. — The effect of PcTX1 on ASIC currents in cortical neurons. Representative traces and statistical results showing PcTX1 (10 nM) markedly decreased the peak amplitude of ASIC current from 815.62 ± 58.64 to 435.33 ± 61.67 pA. After washout, ASIC currents returned to the control level. Data are expressed as means ± SEM. n = 6, # p < 0.05 vs control and *p < 0.05 vs PcTX1. (PDF 99 kb) [file 12974_2015_465_MOESM2_ESM.pdf]

Additional file 2

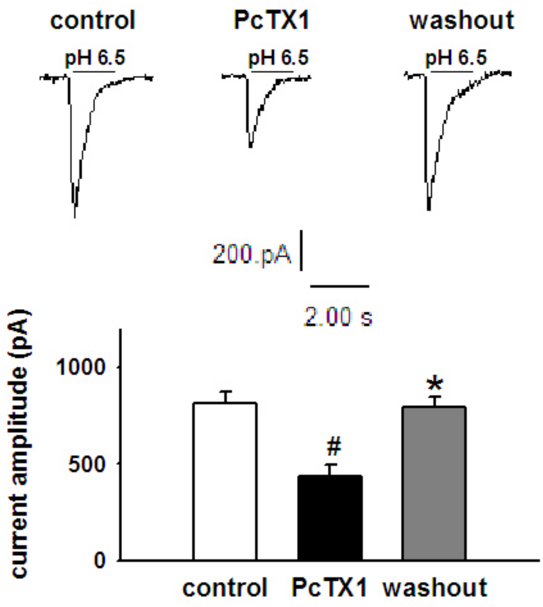

SFig2.. The effect of PcTX1 on ASICs currents in cortical neurons. Representative traces and statistical results showing PcTX1 (10 nM) markedly decreased the peak amplitude of ASIC current from 815.62±58.64 pA to 435.33±61.67 pA. After wash out, ASICs currents returned to the control level. Data are expressed as means ± SEM. n=6, $^{\#}p < 0.05$ vs control and $^{*}p < 0.05$ vs PCTX1.
