# Supplementary material for: Acid-sensing ion channel 1a contributes to the effect of extracellular acidosis on NLRP1 inflammasome activation in cortical neurons
Source: J Neuroinflammation. 2015 Dec 30;12:246. doi: 10.1186/s12974-015-0465-7 (PMC4696203; doi:10.1186/s12974-015-0465-7)
Supplement: Additional file 3: Figure S3. — The effect of pH 6.0 extracellular medium on BK currents and [K+]i in cortical neurons. Representative traces and statistical results showing pH 6.0 extracellular medium increased BK currents from 1.29 ± 0.1 to 1.99 ± 0.19 nA (A) and reduced [K+]i to 65.16 ± 5.77 % (B). Data are expressed as means ± SEM. n = 6, # p < 0.05 vs control and *p < 0.05 vs pH 6.0. (PDF 155 kb) [file 12974_2015_465_MOESM3_ESM.pdf]

## Additional file 3

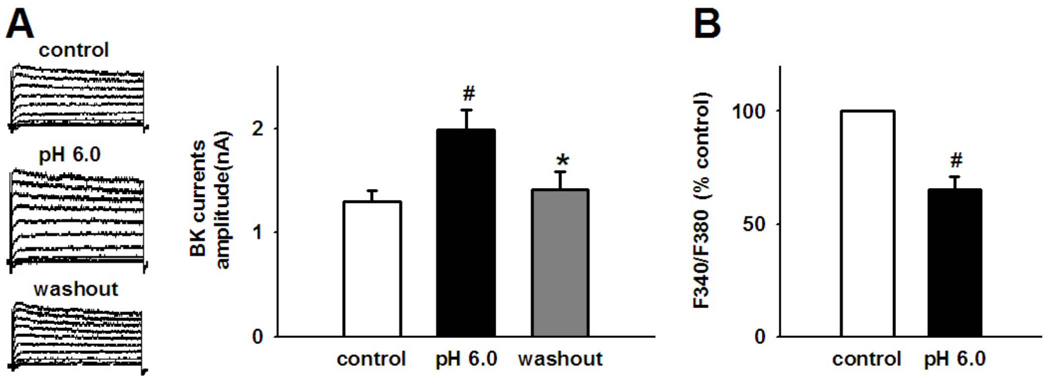


**SFig3. The effect of pH 6.0 extracellular medium on BK currents and $[K^+]_i$ in cortical neurons.** Representative traces and statistical results showing pH 6.0 extracellular medium increased BK currents from 1.29 ± 0.1 nA to 1.99 ± 0.19 nA (A) and reduced $[K^+]_i$ to 65.16 ± 5.77 % (B). Data are expressed as means ± SEM. n=6, $^{\#}p < 0.05$ vs control and $^{*}p < 0.05$ vs pH 6.0.
